# Leaf-Like Sepals Induced by Ectopic Expression of a *SHORT VEGETATIVE PHASE* (*SVP*)-Like MADS-Box Gene from the Basal Eudicot *Epimedium sagittatum*

**DOI:** 10.3389/fpls.2016.01461

**Published:** 2016-09-28

**Authors:** Zhineng Li, Shaohua Zeng, Yanbang Li, Mingyang Li, Erik Souer

**Affiliations:** ^1^Chongqing Engineering Research Center for Floriculture, Key Laboratory of Horticulture Science for Southern Mountainous Regions of Ministry of Education, College of Horticulture and Landscape Achitecture, Southwest UniversityChongqing, China; ^2^Guangdong Provincial Key Laboratory of Applied Botany, South China Botanical Garden, The Chinese Academy of SciencesGuangzhou, China; ^3^Institute for Molecular Cell Biology, Graduate School of Experimental Plant Sciences, Vrije Universiteit AmsterdamAmsterdam, Netherlands

**Keywords:** leaf-like sepal, floral reversion, floral transition, *SVP*, *Epimedium sagittatum*

## Abstract

*Epimedium* L. (Berberidaceae, Ranales), a perennial traditional Chinese medicinal herb, has become a new popular landscape plant for ground cover and pot culture in many countries based on its excellent ornamental characteristics and, distinctive and diverse floral morphology. However, little is known about the molecular genetics of flower development in *Epimedium sagittatum*. Here, we describe the characterization of *EsSVP* that encodes a protein sharing 68, 54, and 35% similarity with SVP, AGAMOUS-like 24 (AGL24) and SUPPRESSOR OF OVEREXPRESSION OF CONSTANS 1 (SOC1) in *Arabidopsis*, respectively. Quantitative RT-PCR (qRT-PCR) indicated that *EsSVP* transcripts were principally found in petiole and leaf tissues, with little expression in roots and flowers and no in fruits. The highest *EsSVP* expression was observed in leaves. The flowering time of *35S::EsSVP* in most *Arabidopsis thaliana* and in all petunia plants was not affected in both photoperiod conditions, but *35S::EsSVP* 5# and *35S::EsSVP* 1# *Arabidopsis* lines induced late and early flowering under long day (LD, 14 h light/10 h dark) and short day (SD, 10 h light/14 h dark) conditions, respectively. The *35S::EsSVP Arabidopsis* produced extra secondary inflorescence or floral meristems in the axils of the leaf-like sepals with excrescent trichomes, and leaf-like sepals not able to enclose the inner three whorls completely. Moreover, almost all transgenic *Arabidopsis* plants showed persistent sepals around the completely matured fruits. Upon ectopic expression of *35S::EsSVP* in *Petunia* W115, sepals were enlarged, sometimes to the size of leaves; corollas were greenish and did not fully open. These results suggest that *EsSVP* is involved in inflorescence meristem identity and flowering time regulation in some conditions. Although, the SVP homologs might have suffered functional diversification among diverse species between core and basal eudicots, the protein functions are conserved between *Arabidopsis*/*Petunia* and *Epimedium*.

## Introduction

The ‘photoperiodic,’ ‘autonomous,’ ‘vernalization,’ and ‘gibberellin’ pathways in *Arabidopsis* seem to focus on the transcriptional regulation of the floral integrator genes *FLOWERING LOCUS T* (*FT*) and *SOC1*, which accelerate expression of *APETALA1* (*AP1*) and *LEAFY* (*LFY*), demanded to confer floral identity on developing floral primordia (Huala and Sussex, 1992; Weigel et al., 1992; Bowman et al., 1993; Mandel and Yanofsky, 1995; Ferrandiz et al., 2000; Corbesier and Coupland, 2005, 2006). FLOWERING LOCUS C (FLC) and SVP restrict the expression of the mobile floral inducer (‘florigen’) *FLOWERING LOCUS T* (*FT*) and other genes that initiate floral transition, in a partly tissue-specific fashion (Searle et al., 2006; Li et al., 2008; Jang et al., 2009). During the early stages of flower development, *AP1* can interact with *SVP* and this complex may initially repress homeotic gene activity. During this stage, also SOC1 blocks the premature expression of floral homeotic B-, C- and E-class genes in inflorescence meristems (IMs) and early floral meristems (FMs) in a redundant manner with AGL24 and SVP, respectively (Gregis et al., 2009; Liu et al., 2009; Torti et al., 2012). Floral integrators (FT and SOC1), floral repressors (SVP and FLC), and a microRNA, targeting AP2-like factors (miR172a), were identified as possible AGL15 targets (Zheng et al., 2009). AGL15 and AGL18, along with SVP and AGL24, are necessary to repress initiation of floral programs in vegetative organs (Fernandez et al., 2014). Members of the *SVP-like* gene family have been ascertained in a great range of species and have been shown to perform diverse functions. In *Arabidopsis*, two paralogous genes, *SVP* and *AGL24*, perform opposite functions during the floral transition (Hartmann et al., 2000; Yu et al., 2002), and have redundant functions during early stages of flower development (Gregis et al., 2006, 2008).

Based on the position where flowers and branches are formed, inflorescences are classified into three broad types: racemes, cymes and panicles (Rickett, 1944; Weberling, 1989; Prusinkiewicz et al., 2007). *Arabidopsis* and *Antirrhinum* produce racemes, whereas petunia produces a cyme. *Epimedium sagittatum*, a member of the basal eudicots belongs to Ranunculales and produces panicles.

*Epimedium* plants are regarded as an excellent evolutionary model for their distinctive and diverse floral morphologies, displaying evolutionarily intermediate forms including petaloid sepals and petals with nectariferous (nectar secreting) tissue on their inner face (Stearn, 2002). Another member of basal eudicots *Aquilegia* (Ranunculaceae, Ranunculales) has been studied as a new model in plant development, ecology, and evolution (Kramer, 2009), but little is known about *SVP* genes in *Aquilegia*. In the past decade, the signaling pathways that promotes flowering and the way floral architecture is determined has been described in *Arabidopsi*s, a member of the core eudicots (Blázquez, 2000; Blázquez, 2005), but whether a similar gene network works in basal eudicots *Epimedium* L. remains a challenging subject in the field of plant molecular development. What is the difference between basal and core eudicots with regard to flower development leading to diverse inflorescences types? To understand the relationship between MADS-box genes and floral organ development in *Epimedium*, we previously isolated and characterized homologs of the *AP1*/*SEPALLATA*(*SEP*)/*AGL6* superclade of MADS-box genes in *E. sagittatum* (Sun et al., 2014). Here, we isolated an *SVP*-like MADS-box gene from *E. sagittatum*. Transgenic approaches and transcriptional analysis were used to further investigate the potential role of *EsSVP*, pointing at an important role in inflorescence meristem development and flowering time regulation.

## Materials and Methods

### Plant Material and Growth Condition

Plant material for gene cloning and qRT-PCR analysis were obtained from *E. sagittatum*. The samples were collected from individuals in the experimental field of Wuhan Botanical Garden, the Chinese Academy of Sciences that were originally introduced from Hunan province, China. Leaves, petioles, flower buds, flowers, and roots were sampled and immediately frozen in liquid nitrogen and kept at -70°C until required. Total RNA from flower buds and flowers was isolated for *EsSVP* cloning.

Overexpression of *EsSVP* was carried out in the *Arabidopsis* Columbia-0 (Col-0) ecotype. The *svp-41* lines used for complementation were screened out by its phenotype combined with RT-PCR. The Col-0, *svp-41* and transgenic *Arabidopsis* plants were grown at 22°C under LD and/or SD conditions. To investigate flowering time, the number of rosette and/or cauline leaves of transgenic plants T3 were counted when plants bore a 1-cm-long inflorescence. Before the first flower formation, the transgenic *Arabidopsis* seedlings were sampled for qRT-PCR. Wild type *Petunia* W115 and *extrapetals* (*exp^W2115^*) was grown at average 22°C under normal greenhouse conditions. The *Petunia* mutant *exp^W2115^*, containing an insertion of a *dTph1* transposon in an *SVP*-like gene, arose spontaneously among progeny of the line W138 (Castel, 2009). The *exp^W2115^* plants used for functional complementation were screened out by phenotype, which is characterized by an inflorescence with a single terminal flower compared with successive terminal flowers in the wild type (**Supplementary Figures S1A,B**). Because seasonal changes in day length and/or light intensity might influence plant development and flowering time, care was taken to grow different genotypes side by side under the same conditions for comparison. The transgenic progeny was scored by phenotype and analyzed by RT-PCR.

### Cloning, Sequence Alignment, and Phylogenetic Analysis

The fragment of *EsSVP* cDNA of *E. sagittatum* was obtained by 454 GS-FLX pyrosequencing technology (Zeng et al., 2010). Cloning of the full-length *EsSVP* gene was accomplished by using a 3′/5′-RACE (rapid amplification of cDNA ends) strategy as previously reported (Li et al., 2012), using GSP5-3 and GSP3-5 to amplify the 3′ and 5′ terminal regions of *SVP*-orthologous gene, respectively. Universal 3′ and 5′ PCR primers were supplied by the SMART^TM^ cDNA Library Construction Kit (Clontech, Palo Alto, CA, USA). For vector construction, the complete cDNA sequence of *EsSVP* was obtained by primer pairs *EsSVP*-V-F and *EsSVP*-V-R (**Supplementary Table S1**; **Supplementary Figure S2**). The sequences of selected species were downloaded from the NCBI GenBank^1^ Multiple alignment of predicted amino acid sequences were generated using Clustal W 1.83 (Thompson et al., 1997) with a gap open penalty of 10.00 and a gap extension penalty of 0.05. Neighbor-joining (NJ) bootstrap analysis (1,000 replications) with the maximum composite likelihood model for the DNAs and the Poisson correction for the amino-acids were performed by MEGA 4 (Tamura et al., 2007). During the analyses, *Arabidopsis* SOC1 was used as an out-group. Sequence data from this article can be found in the GenBank/EMBL databases under the following accession numbers: SVP (NM_127820), AGL24 (NM_118587) and SOC1 (NM_130128) in *Arabidopsis*, potato StMADS11 (AF008652), StMADS16 (AY643736), rice OsMADS22 (AB107957), OsMADS47 (AY345221), OsMADS55 (AY345223), barley HvBM1 (AJ249142), HvBM10 (EF043040), AfSVP.1 (HQ173338) and AfSVP.2 (HQ173339) in *Aquilegia formosa*, MpMADS1 (AB050643) in *Magnolia praecocissima* and DlSVP (KM657947) in *Dimocarpus longan*.

### Construction of *EsSVP*-Overexpression Lines

For *35S:EsSVP*, the *EsSVP* cDNA including partial 5′ and 3′- untranslated regions (UTRs) was amplified using the primer pairs *EsSVP*-V-F and *EsSVP*-V-R (**Supplementary Table S1**; **Supplementary Figure S2**) with *Pfu* DNA Polymerase (Stratagene, La Jolla, CA, USA) and cloned in pMD18-T Vector. Sequence accuracy and insertion direction were confirmed by sequencing. After digestion with *Sal*I and *Sac*I restriction enzymes, the insert was ligated into the modified binary vector pBI121 containing the CaMV*35S* promoter and the *Nos* terminator. The construct was transformed to *Agrobacterium tumefaciens* strain GV3101 or EHA105 and used to transform *Arabidopsis* by the floral dip method (Clough and Bent, 1998) or *Petunia* via leaf disk transformation (Horsch et al., 1985). Data shown are representative phenotypes based on the analysis of multiple independent transformants.

### Analysis of Gene Expression

For expression analysis, total RNA was prepared using Trizol REAGENT (Invitrogen, Carlsbad, CA, USA) according to the manufacturer’s instructions. RQ1 RNase-Free DNase (Promega, USA) pre-treatment of the RNA samples was applied to eliminate all non-RT-dependent background. 3 μg of DNase pre-treated total RNA was reverse transcribed in a total volume of 20 μL with 0.5 μg oligo(dT)15, 0.5 mM dNTPs, 10 mM DTT, 40 U RNasin^TM^ Ribonuclease Inhibitor (Promega, USA) and 200 U SuperScript II RNase H- reverse transcriptase (Invitrogen, Carlsbad, CA, USA).

To determine the expression pattern of *SVP* and/or *EsSVP* in transgenic *Arabidopsis* and/or *Petunia*, RT and/or qRT-PCR was performed. The qRT-PCR assays of putative genes including *CO*, *FT*, *FLC*, *SVP*, *SOC1*, *LFY*, and *AP1* were performed as described below. The primers for RT and qRT-PCR are listed in **Supplementary Table S1**. Reactions were performed with the SYBR Premix Ex Taq (Bio-Rad, USA) and analyzed in Eco^TM^ Real-Time PCR System (USA). Thermocycler conditions were 2 min at 50°C followed by 10 s at 95°C and 40 cycles of 15 s at 95°C, 20 s at 60°C, and 30 s at 72°C. Relative amounts of transcripts were calculated by the comparative CT (threshold cycle number at the cross-point between amplification plot and threshold) method, and values were normalized. The *Actin* homolog of *E. sagittatum* (Huang et al., 2015) and the *Tubulin2* (β-tubulin) gene of *Arabidopsis* (AY054693) were used as internal controls. The qRT-PCR products were amplified using 4 μl of the RT reaction mixture, 5 μl 2×SYBR Green Master Mix, 0.5 μl forward and reverse primer (10 μmol/μl) and water to a final volume of 10 μl. RT-PCR conditions were 94°C for 1 min, followed by 28 cycles at 94°C for 30 s, 58°C for 30 s, 72°C for 1 min, and a final extension of 72°C for 3 min. The qRT-PCR was performed in three replicates for each sample and data are shown as mean values ± SD (standard deviation).

### Histological Section

Floral buds and open flowers were fixed in EAF buffer (50% Ethanol, 0.5% Acetic Acid, 3.7% Formaldehyde) by applying vacuum for 15 min, then incubate 1 h at 4°C in EAF. Vacuum and incubation steps were repeated for three times. For serial microtome sections, FMs were embedded in paraffine (Paraplast Plus) after dehydration and penetration of paraffine into the plant material. The 8-mm-thick sections were stained with toluidine blue for 10 min and rinsed by deionized water for 5 min, then eosin for 5 min and dehydration by 95 and 100% ethanol for three times 15 min, deparaffine the sections in xylene.

## Results

### Sequence Alignment and Phylogenetic Analysis

**FIGURE 1:**
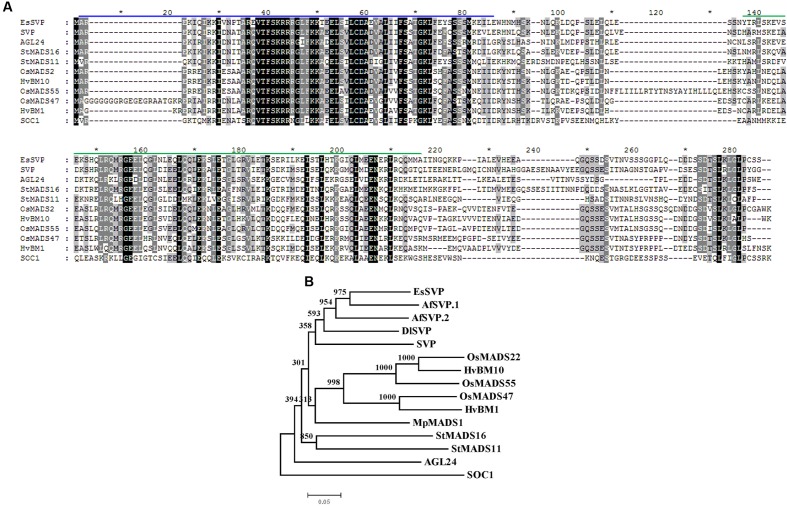
**Comparison of predicted SVP-like protein EsSVP sequences.** **(A)** Alignment of *Epimedium sagittatum SVP*-like amino acid sequences with *Arabidopsis SVP*, *AGL24*, *SOC1*, and potato *StMADS11*, *StMADS16*, and barley *HvBM1*, *HvBM10*, and rice *OsMADS22*, *OsMADS47*, *OsMADS55*. The amino acid residues in the alignment are colored according to the following scheme: white on black, identical or conservative residue; white on gray, similar residues; black on white, non-similar or weakly similar residues. Black boxes indicate fully conserved residues; shaded boxes indicate similar and partially conserved residues. A MEF2-like motif spanning amino acids 1–58 in the N-terminus and a K-box motif in the middle are overlined in blue and green. **(B)** Phylogenetic tree based on the amino acid datasets with MIKC domains alignment of *EsSVP* in *Epimedium* and *SVP*-like from other plant species. The tree was generated with MEGA4.1 software, using the Neighbor-Joining method and 1000 bootstrap replicates. The scale bar indicates a divergence of 0.05 amino acid substitutions per site. SVP (NM_127820), AGL24(NM_118587) and SOC1 (NM_130128) in *Arabidopsis*, potato StMADS11 (AF008652), StMADS16 (AY643736), rice OsMADS22 (AB107957), OsMADS47 (AY345221), OsMADS55 (AY345223), barley HvBM1 (AJ249142), HvBM10 (EF043040), AfSVP.1 (HQ173338) and AfSVP.2 (HQ173339) in *Aquilegia formosa*, MpMADS1 (AB050643) in *Magnolia praecocissima* and DlSVP (KM657947) in *Dimocarpus longan*. SOC1 was used as an out-group.

The 1027 bp length *EsSVP* mRNA contains an open reading frame (ORF) of 678 bp with 5′ and 3′- UTRs of 87 and 241 bp respectively (GenBank accession no. KX250266, **Supplementary Figure S2**). The predicted EsSVP protein of 226 amino acid residues contains a MEF2-like motif in the N-terminus and a K-box motif in the middle (**Figure 1A**). The putative EsSVP protein shares high similarity with other SVP-related proteins, e.g., it shows 68, 54, and 35% identity with the products of the *SVP*, *AGL24*, and *SOC1* in *Arabidopsis*, respectively (**Figure 1A**). Sequence comparison of the putative protein sequence with other published MADS-domain proteins indicates that EsSVP shows extensive similarity to *StMADS11* and *StMADS16*-like proteins (around 52 and 55%, respectively).

To better understand the evolutionary relationships, phylogenetic analysis of EsSVP with the *SVP*/*StMADS11*-like genes from other plant species was performed. EsSVP clustered closely to AfSVP.1 and AfSVP.2 from *Aquilegia formosa*, DlSVP from *Dimocarpus longan*, SVP from *Arabidopsis* (Hartmann et al., 2000), OsMADS22, OsMADS47 and OsMADS55 from rice (Sentoku et al., 2005), HvBM1 and HvBM10 from barley (Trevaskis et al., 2007), and MpMADS1 from *Magnolia praecocissima.* They all cluster in the SVP/StMADS11-like group (Carmona et al., 1998; **Figure 1B**). This shows that EsSVP belongs to the SVP group, which includes the SVP homologs (*StMADS11-*like) from *Arabidopsis*. This group is distinct from *Arabidopsis* AGL24 and SOC1 proteins (**Figure 1B**). Thus, EsSVP appears to be an *SVP*/*StMADS11*-like transcription factor.

### Expression Pattern of *EsSVP* in *E. sagittatum*

**FIGURE 2:**
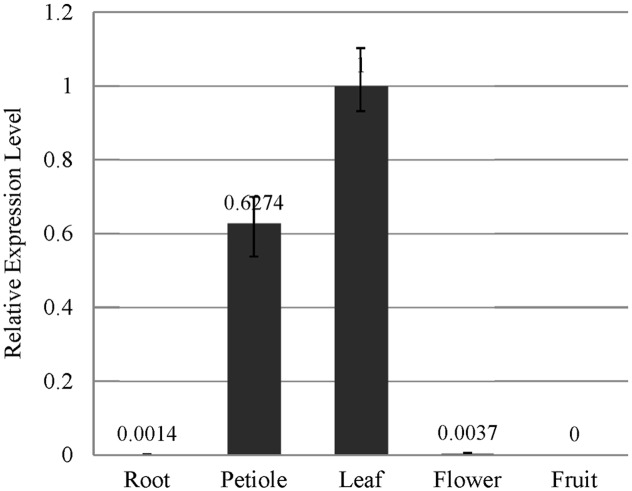
***EsSVP* expression analysis in *E. sagittatum* roots, petioles, leaves, flowers and fruits by qRT-PCR.** Error bars represent standard deviations calculated from three replications. An *Actin* homologous gene of *E. sagittatum* was used as an internal control.

The results of our qRT-PCR experiment indicated that *EsSVP* transcripts were principally found in leaf tissues and petioles, but hardly in flowers and roots, and no expression was found in fruits of *Epimedium*. The expression of *EsSVP* in flowers is approximately 2.7-fold higher than that in roots. The highest *EsSVP* expression was observed in leaves, reaching nearly 740 and 270-fold higher expression than in roots and flowers respectively, and about 1.6-fold of the petioles expression (**Figure 2**). Based on these results, we conclude that a high level of *EsSVP* expression occurs in leaves, especially in leaf lamina, which might contribute to maintaining vegetative growth.

### Ectopic Expression of *EsSVP* in *Arabidopsis*

**FIGURE 3:**
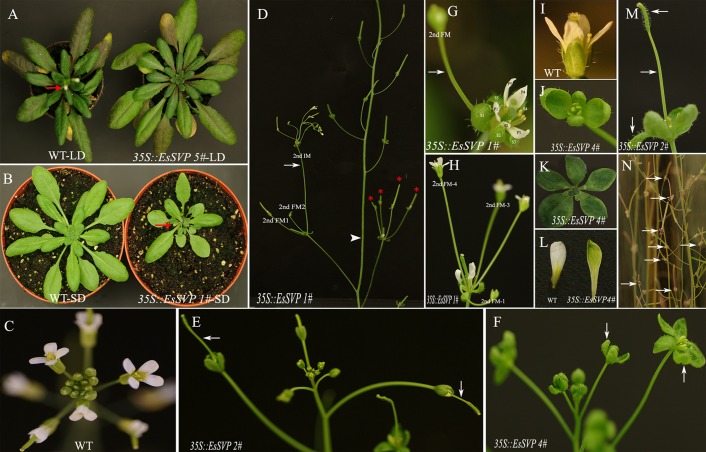
**Phenotypes of *p35S::EsSVP* transgenic *Arabidopsis*.** **(A,B)** Wild type (Columbia-0) (left) and ectopic expression of *35S::EsSVP* in wild type *Arabidopsis* (right). **(A)** *35S::EsSVP* 5# causes late flowering under LD conditions (right), **(B)** *35S::EsSVP* 1# causes early flowering under SD conditions (right). The arrow indicates the first flower. **(C)** Wild type *Arabidopsis* Col-O inflorescence. **(D)** Inflorescence of *35S::EsSVP* 1# showing floral reversions. The arrow indicates a flower that is replaced by an indeterminate racemose shoot; The arrowhead points at the main stem, and the arrow indicates a 2nd IM, red snowflake shows four 2nd FM. **(E)** FM of *35S::EsSVP* 2# can cause excrescent trichomes on fruit with longer fruit stem (arrow). **(F)** FM of *35S::EsSVP* 4# leaf-like sepal, green sepaloid petals compared with empty vector control inflorescence **(C)**. **(G)** Trichomes on the adaxial side of the leaf-like sepals. Sepals are not able to enclose the inner developing organs completely. Five sepals and petals produced in one of the primary FMs (arrow). **(H)** Four extra flowers develop next to carpel, with excrescent trichomes on leaf-like sepals. **(I)** Wild type *Arabidopsis* Col-O flower. **(J–L)** Close inspection of *35S::EsSVP* 4# flowers and sepals. Four **(J)** and five **(K)** green sepaloid petals compared with empty vector control flower **(I)**. Green sepaloid petals (**L**, right) compared to the normal white ones in wild type. **(M)** Close inspection of trichomes on fruit, longer fruit stem, leaf-like sepals (arrow) in *35S::EsSVP* 2# flower. **(N)** Persistent sepals (arrows) around the matured fruits.

Thirty independently transformed *Arabidopsis* plants containing the full-length sense *EsSVP* cDNA driven by the 35S promoter were generated (*35S*::*EsSVP Arabidopsis*). The presence of the transgene in these lines was confirmed by PCR on genomic DNA. Twelve of the transformed *Arabidopsis* T3 lines showed various degrees of phenotypic alterations in their reproductive organs, as compared to wild type plants. Ectopic expression of *35S::EsSVP* caused floral reversion with the central primary flower forming extra secondary inflorescence meristems (2nd IM) and secondary floral meristems (2nd FM) in the axils of the leaf-like sepals (**Figure 3D**), and also excrescent trichomes on leaf-like sepals and fruits (**Figures 3G,J,K,M**). Based on the total number of cauline and/or rosette leaves, *35S::EsSVP* 5# *Arabidopsis/* Col-0 plants showed late flowering under LD conditions (**Figure 3A**), while *35S::EsSVP* 1# lines showed early flowering under SD conditions (**Figure 3B**; **Table 1**). *35S::EsSVP* 1# *Arabidopsis* generated a central primary flower with an extra one, two or four 2nd FMs and one 2nd IM in the axils of leaf-like sepals. Often these extra flowers reiterated this pattern producing a highly branched floral structure (**Figures 3D,H**). Five sepals and petals produced in one of the primary FMs (**Figure 3G**). *35S::EsSVP* 1#, 2#, and 4# generate leaf-like sepals that develop trichomes on their adaxial side and these leaf-like sepals are not able to enclose the inner developing organs completely (**Figures 3D–J**). *35S::EsSVP* 2# *Arabidopsis* developed excrescent trichomes on fruits (**Figures 3E,M**). *35S::EsSVP* 4# produced four or five leaf-like sepals (**Figures 3J,K**) and green sepaloid petals (**Figures 3F,K,L**), comparing with empty vector control (**Figures 3C,I,L**). In general, transgenic *Arabidopsis* showed persistent sepals around the completely matured fruit (**Figure 3N**). Apart from that, no morphological or microstructure alterations were detected in the third and fourth whorl organs. After kanamycin-resistance selection combined with RT-PCR screening, five *35S::EsSVP*/*svp-41* transformants were obtained, but none showed complementation of the mutant phenotype (data not shown).

**Table 1:** Effect of overexpression of *EsSVP* on flowering time as determined by leaf numbers under long/short day conditions in *Arabidopsis*.

Light condition	Genotype	Rosette leaf numbers^a^	Cauline leaf numbers^b^	Total numbers of leaves^c^
LD	*35S::EsSVP LD5#*	26.12 ± 3.20 ^∗∗^	5.59 ± 1.12^∗∗^	31.71 ± 3.51^∗∗^
	WT	11.00 ± 0.79	2.24 ± 0.44	13.24 ± 1.03
SD	*35S::EsSVP SD1#*	9.44 ± 0.89 ^∗∗^	1.44 ± 0.51 ^∗∗^	10.88 ± 1.31^∗∗^
	WT	53.94 ± 1.18	10.06 ± 1.00	64.00 ± 1.03


*^∗∗^Significance at 1% level (*P*-value < 0.01) compared with wild type *Arabidopsis*. ^a,b,c^ Average numbers ± SD. Twenty-eight *35S::EsSVP LD5#* and 31 *35S::EsSVP SD1#* transgenic plants were used for observation under LD and SD condition, respectively. Thirty WT *Arabidopsis* plants were used as a control under LD/SD condition.*

**FIGURE 4:**
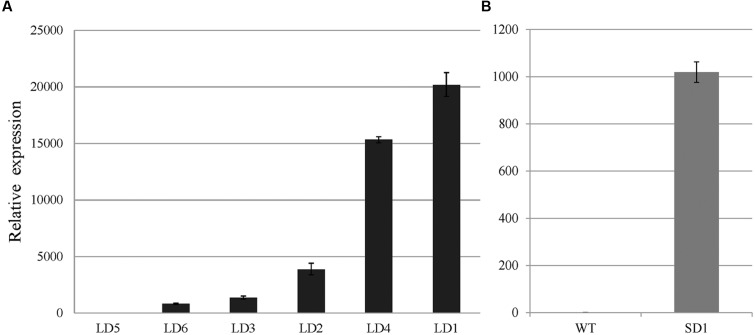
***EsSVP* relative expression in leaves of *35S::EsSVP Arabidopsis* lines.** Different lines under LD condition **(A)** and *1#* under SD condition **(B)**, with the *TUBULIN2* gene as an internal control. Error bars indicate the SD of the average of relative mRNA amounts determined as triplicates in two independent biological replicates.

**FIGURE 5:**
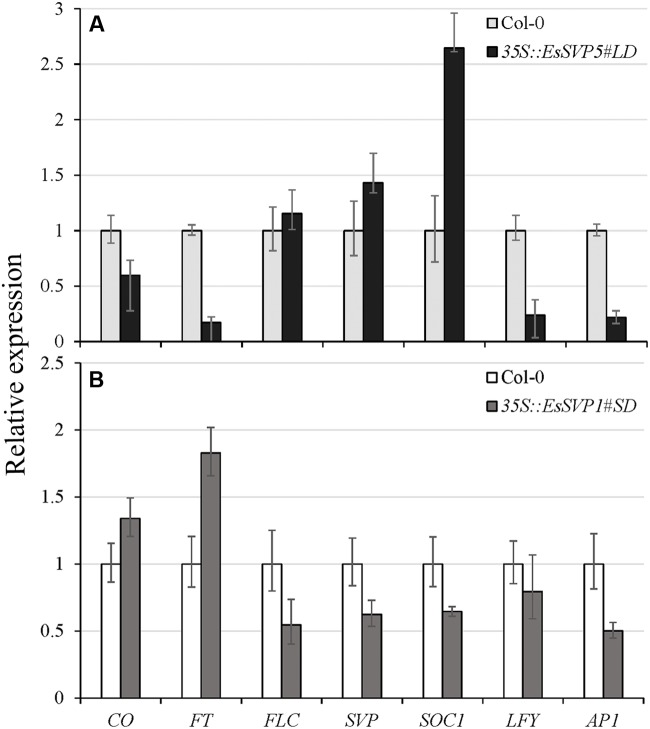
**Relative expression levels of different flowering time genes (*CO*, *FT*, *FLC*, *SVP*, *SOC1*, *LFY*, and *AP1*) in *35S::EsSVP* 5#LD and 1#SD transgenic *Arabidopsis* as determined by qRT-PCR, with the *TUBULIN2* gene as an internal control.** *35S::EsSVP* 5#LD **(A)** and 1#SD **(B)** under LD and SD condition, respectively. Error bars indicate the SD of the average of relative mRNA amounts determined as triplicates in two independent biological replicates.

*SVP* and *EsSVP* expression levels in mature leaves of *35S::EsSVP* transformants were investigated by qRT-PCR analysis (for T_3_ generation plants in *35S::EsSVP* SD1*# Arabidopsis*, the plant just as **Figure 3B**). The relative expression of *EsSVP* reached over 1000-fold the background level (**Figure 4B**), while *SVP* was expressed at slightly lower levels, that is just 0.63-fold of the Col-0 (**Figure 5B**). *EsSVP* expression levels in seedlings of *Arabidopsis* transgenic plants varied among different lines. Relative *EsSVP* expression levels in *35S::EsSVP* LD 2#, 4# were approximately 2.8 and 11-fold higher compared to LD 3#, respectively. The highest *EsSVP* expression occurred in *35S::EsSVP* LD 1#, reaching nearly 14.8-fold the LD 3#. The relative *EsSVP* expression in *35S::EsSVP* LD 5# is the lowest among the transgenic lines. The relative expression of *EsSVP* gene in LD 1#, 4#, and 2# is nearly 20-, 15- and 3.8-1000-fold the expression found in the LD 5# (**Figure 4A**).

In most *A. thaliana EsSVP* transformants, the flowering time was not affected under both photoperiod conditions, but *35S::EsSVP* 5# and *35S::EsSVP* 1# *Arabidopsis* lines induced late and early flowering under LD and SD conditions, respectively. In the *35S::EsSVP Arabidopsis* seedlings, *CO* and *FT* transcripts were significantly downregulated, but *FLC*, *SVP* and *SOC1* were upregulated in *35S::EsSVP* 5#LD comparing to Col-0, which showed late flowering under LD conditions (**Figures 3** and **5A**). On average, the total number of leaves at flowering was 31.7 compared to 13.2 in Col-0 (**Table 1**) showing late flowering, whereas floral organs developed normally (**Figure 3**). However, in the early flowering *35S::EsSVP* 1#SD *Arabidopsis* line under SD conditions, *CO* and *FT* transcripts were significantly upregulated, but *FLC*, *SVP* and *SOC1* were downregulated to different degrees (**Figure 5B**). The total number of leaves at flowering was 10.9 compared to 64 in Col-0 on average (**Table 1**).

### Ectopic Expression of *EsSVP* in *Petunia*

**FIGURE 6:**
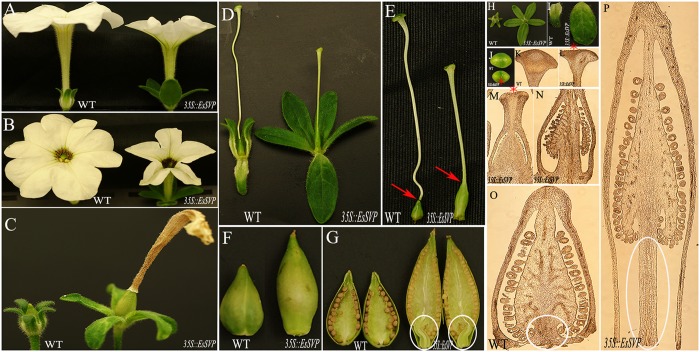
**Phenotypes of the *35S::EsSVP 25#* transgenic *Petunia* line compared with wild type W115 (WT).** **(A,B)** Side and top view of *35S::EsSVP* flower (right) compared with WT (left). Sepals are enlarged, petals are greenish and flowers do not fully open. **(C)** Leaf-like sepals and enlarged fruit with persistent petal (right) compared with WT (left). **(D,H)** Close inspection of five not enclosed leaf-like sepals. **(E)** Close inspection of an abnormal pistil. In the *35S::EsSVP* transformant, a shortened style is not easily separated from elongated and enlarged ovaries (arrows). **(F,G)** External **(F)** and vertical section **(G)** showing fruit morphology. *35S::EsSVP* fruit appears longer and narrower, the base of the placenta elongated (circle shown). **(I)** Close inspection of single sepal. **(J)** Top view and paraffin section of stigma with a sunken surface on the top (red asterisk). **(K–P)** Paraffin section of different stages of pistil **(K,O)** Wild type stigma and ovary. **(L,P)** *35S::EsSVP* line 25# stigma and ovary. In **(P)** a stem-like structure at the base of the septum inside the elongated ovaries is seen (circled). **(M)** Young and short pistil in *35S::EsSVP* with a sunken surface on the top of the stigma (red asterisk). **(N)** Ovary elongation in *35S::EsSVP* **(N)**. Magnification in K-P: 2.5 × 10.

Forty-two independent *Petunia* transformants containing a *35S::EsSVP* transgene were generated, which were all confirmed by PCR of genomic DNA. Twelve of the transgenic *Petunia* lines showed various degrees of phenotypic alteration in the reproductive organs, as compared to wild type Petunia W115 plants. Expression of *35S::EsSVP* in wild type *Petunia* W115 had little or no effect on flowering time and did not affect cymose branching, but clearly affected flower development. That is, the five separated sepals were enlarged, sometimes to the size of leaves. Furthermore, these leaf-like organs continued to expand during flower development and after anthesis (**Figures 6D,H,I**). The corollas were greenish and did not fully open (partial loss of petal identity; **Figures 6A,B**). The shortened style was not easily separated from the elongated and enlarged ovary (arrow shown, **Figures 6E,N,P**). A sunken surface on the top of the stigma appeared compared with wild type (red asterisk shown, **Figures 6J–M**). The base of the placenta elongated many fold (**Figure 6P**, paraffin section material just in **Figure 6E**, right) compared with WT (**Figure 6O**, paraffin section material just in **Figure 6E**, left). *35S*::*EsSVP* fruits (**Figure 6F**), appears longer and narrower and with the base of the placenta elongated (**Figure 6G**, circled). *EsSVP* gene overexpression in *Petunia* resulted in a range of floral reversion phenotypes, but was not able to phenotypically complement a *Petunia exp* mutant, which is characterized by an inflorescence with a single terminal flower compared with successive terminal flowers in the wild type and *35S::EsSVP* inflorescences (**Supplementary Figures S1A,B,C**).

**FIGURE 7:**
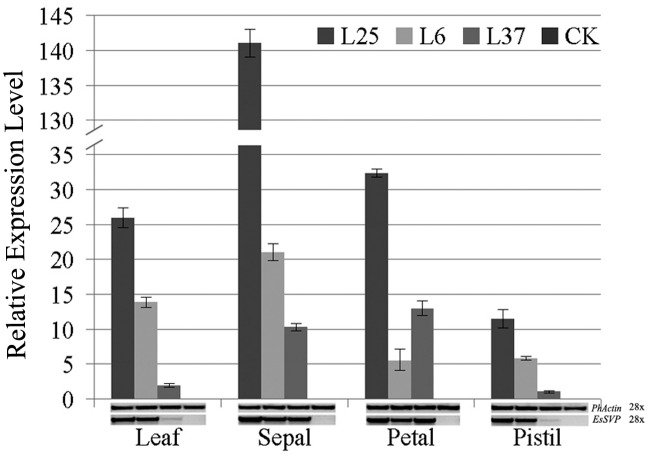
**RT and qRT-PCR on leaves, sepals, petals and pistils of *35S::EsSVP Petunia*.** Error bars represent standard deviations calculated from three replications. The *PhActin* homolog gene of *Petunia* was used as an internal control. The number of PCR cycles is indicated next to the gene names. Different transgenic lines (L25, strong; L6, medium; L37, weak) were selected according to the strength of phenotype. WT indicates wild type W115.

To examine the expression profile of *EsSVP* in transgenic *Petunia*, RT- and qRT-PCR was carried out using cDNA derived from mature leaves, sepals, petals, and pistils in Line25 (L25), L6 and L37 with strong, medium and weak phenotypes. Relative expression in leaves, petals or pistils in L25 is higher than detected in L6, and that of L6 is higher than detected in L37, respectively. The relative expression in petals in L37 is >2.3 -fold higher than detected in L6. Relative expression in leaves in L25 is approximately 1.9 and 13.5-fold higher expression levels than detected in L6 and L37, respectively. Relative expression levels reached a peak in L25 in sepals (i.e., approximately 4.4, 5.4, and 12.3-fold higher expression levels than detected in petals, leaves, and pistils, respectively), reaching >6.7 and 13.7-fold higher expression level than detected in L6 and L37, respectively (**Figure 7**).

These results suggest that ectopic *EsSVP* expression in *Petunia* resulted in leaf-like sepals, not fully opened greenish corollas, and abnormal stigma and matured ovary.

## Discussion

### Evolution of *EsSVP* and its Expression Profile

In early diverging eudicots, the basal eudicots (Buxaceae, Trochodendrales, Proteales, Sabiaceae, and Ranunculales) form separate well-supported clades, which are clustered closely to monocots and Chloranthaceae (Li et al., 2012). *Epimedium* belongs to Berberidaceae, Ranunculales, and is a member of the basal eudicots. Phylogenetic analysis showed that EsSVP was clustered closely to AfSVP.1 and AfSVP.2 in *Aquilegia formosa*. All these genes clustered in the SVP/ StMADS11-like group which have similar functions in promoting vegetative growth or suppressing flowering (Li et al., 2010). According to phylogenetic analysis, which included *AGL24*, a MADS-box gene most similar to *SVP* in *Arabidopsis* (Gregis et al., 2006), *EsSVP* was more closely related to *SVP.* These findings suggest that the EsSVP MADS-box protein in *Epimedium* might be orthologous to the *Arabidopsis SVP* gene.

In the past, members of the *SVP-like* gene family performing diverse functions have been identified in a range of species. In *Arabidopsis*, *SVP* is expressed during early stages of flower development together with *AGL24* (SVP until stage 3; Hartmann et al., 2000). Both *SVP* and petunia *exp* are expressed during vegetative growth throughout the shoot apical meristem, in leaf primordia and in the veins of leaves (Hartmann et al., 2000; Li et al., 2008; Castel, 2009). *SVP* is transiently expressed in lateral (floral) primordia, but absent from developing flowers and siliques (Hartmann et al., 2000; Castel, 2009). *INCOMPOSITA* (*INCO*) in *Antirrhinum* is transiently expressed in reproductive meristems (Masiero et al., 2004). Similar to the other *SVP-like* genes, *EsSVP* transcript was expressed intensively in the vegetative stage, as shown here. We found *EsSVP* expression in petiole and leaf tissues, with little in root and flower and no expression in fruit. The highest *EsSVP* expression was observed in leaves, reaching over 270-fold the flower level.

### High Expression of *EsSVP* Resulted in Leaf-Like Sepals and Abnormal Flowers

Interestingly, late flowering, floral reversions and shoot-like flowers were induced by overexpression of *SVP* or *INCO* in transgenic *Arabidopsis*. This suggests that they could either directly repress the vegetative to floral transition in *Arabidopsis*, or could indirectly interfere with the function of proteins controlling flowering (Hartmann et al., 2000; Masiero et al., 2004). On the other hand, *INCO* inhibits the prophyll development as an important control gene, which has not been reported yet for MADS-box genes (Masiero et al., 2004). Ectopic expression of *SVP* homologs from rice, barley, Chinese cabbage, Kiwifruit, trifoliate orange, and *Eucalyptus* in *Arabidopsis* causes similar phenotypes, including 2nd IM or 2nd FM in the axils of the leaf-like sepals with excrescent trichomes, leaf-like sepals not able to enclose the inner three whorls (Brill and Watson, 2004; Lee et al., 2007, 2012; Trevaskis et al., 2007; Fornara et al., 2008; Li et al., 2010; Wu et al., 2012).

Constitutive expression of *OsMADS22* and *OsMADS55* led to floral reversion phenotypes including leaf-like sepals, similar to that of the *SVP* floral overexpressor phenotypes, whereas only *OsMADS55* expression induced late flowering arising from the repression of *FT* and *SOC1*. The complementation experiments showed that *OsMADS55*, but not *OsMADS22*, was able to rescue the early flowering phenotype and ambient temperature-insensitive flowering of *svp-32* mutants (Lee et al., 2012). Some *SVP*/*StMADS11*-like genes from herbaceous plants, Chinese cabbage (Lee et al., 2007) and barley (Trevaskis et al., 2007) also act as flowering repressors.

Over-expression and complementation of Kiwifruit *SVP*-like genes in *Arabidopsis* resulted in a range of abnormal floral morphologies via interactions with *Arabidopsis* MADS-box proteins (Wu et al., 2012). Ectopic overexpression of *PtSVP* in *Arabidopsis* resulted in late flowering, additional trichomes and floral defects, such as flower-like structures instead of carpels (Li et al., 2010). The ectopic expression of *EgSVP* in *Arabidopsis* caused a slight delay in flowering time and produced additional inflorescences (Brill and Watson, 2004). Transcription of kiwifruit *SVP1*/*SVP3* or trifoliate orange *PtSVP* in *Arabidopsis svp-41* was able to complement the *svp* mutant (Li et al., 2010; Wu et al., 2012).

We determined conservation of biological function of an *Epimedium* SVP-like gene by over expression and complementation tests performed in *Arabidopsis* and/or *Petunia* and compared the outcome to various experiments in which SVP-like genes from other species were ectopically expressed. *EsSVP* gene over-expression in *Arabidopsis* and *Petunia* resulted in a range of floral reversion phenotypes, but was not able to complement a mutant affected in an *svp*-like gene in *Petunia.* Either, slight differences in these proteins are accountable for this or expression from the 35S promoter does not generate the appropriate expression pattern regarding timing and place.

*SVP* is considered to be a flowering repressor in *Arabidopsis* as it functionally delays flowering time. In this study, flowering time of *35S::EsSVP* transgenic *Arabidopsis* under SD conditions or *Petunia* plants was not delayed. Ectopic expression of *35S::EsSVP* 5# and *35S::EsSVP* 1# in *Arabidopsis* resulted in late flowering under LD conditions and early flowering under SD conditions, respectively (**Figures 3A,B**). The relative *EsSVP* expression level in *35S::EsSVP* 5# was much lower than *35S::EsSVP* 1#, 4#, 2# on LD (**Figure 4A**). Moderate expression of *EsSVP* in *Arabidopsis* indeed delays flowering in *35S::EsSVP* 5# on LD, but *35S::EsSVP* 1#, 4#, and 2# are not affected on flowering time in the same photoperiod conditions. It seems that there is no obvious correlation of the mutant phenotype (number of leaves in *35S::EsSVP* on LD) and the expression levels of *EsSVP*. However, the higher expression levels of *35S::EsSVP* transformants under LD conditions, such as *35S*::*EsSVP* 1#, 4# and 2#, the more obvious phenotype was observed on floral organs variation, including abnormal IMs and FMs from axils of the leaf-like sepals with excrescent trichomes. The relative expression of *SVP* in *35S::EsSVP* 1# line just 0.63-fold of the WT (Col-0; **Figure 5B**). *35S::EsSVP* 1# showing early flowering under SD conditions might thus be a consequence from post-transcriptional gene silencing. Functional divergence of *EsSVP* might have occurred partly because *Epimedium* belongs to the Ranunculales, a member of the basal eudicots.

Recent studies demonstrated that *SVP* and *AGL24* are also floral meristem identity genes. Ectopic *SVP* and *AGL24* expression induces floral meristem indeterminacy by promoting the development of new ectopic floral meristems rather than causing floral reversions (Gregis et al., 2008). Ectopic expression of barley *BM1* and *BM10* (Trevaskis et al., 2007), kiwifruit *SVP*-like (Wu et al., 2012), rice *OsMADS55* (Fornara et al., 2008; Lee et al., 2012) in *Arabidopsis*, and petunia *UNSHAVEN* (*UNS*) and *EXP* overexpression in *Petunia* (Ferrario et al., 2004; Castel, 2009) can give rise to leaf-like sepals. In this study, *EsSVP* induced one to four flower-like structures or an inflorescence in place of the carpel, with fully opened leaf-like sepals and green sepaloid petals (**Figures 3D,G,H**). There are no leaf-like sepals in *E. sagittatum*, but its sepals are always are bigger than petals. Whether EsSVP determines sepal size in *Epimedium* remains to be established. Outer four sepals are purple spotted and apex blunt, outer pair narrowly ovate and inner pair oblong-ovate is ca. 3.5 × 1.5 and 4.5 × 2 mm, respectively; inner sepals are white, ovate-deltoid, apex acute and ca. 4 × 2 mm in size. Petals are brownish yellow, saccate, blunt and 1.5–4 mm in size (Maximowicz, 2011).

More trichomes are usually considered to be a juvenile phenotype of *Arabidopsis* (Telfer et al., 1997). The *35S::EsSVP* lines showed a typical juvenile character, excrescent trichomes on opened leaf-like sepals and fruits. The overexpression of *EsSVP* induced floral reversions and juvenile characteristics during the adult stage, demonstrating that the expression of *SVP* disturbs flower development, acts as a floral repressor similar to *SVP*, and is involved in organ determination.

Constitutive expression of *35S::EsSVP* in petunia W115, caused little or no effect on flowering time and cymose branching, but clearly affected flower development, including not fully opened greenish corollas, not enclosed leaf-like sepals, sometimes to the size of leaves. This result also coincides with the reports on overexpression of the SVP-like gene, *p35S*::*EXP* in wild type *Petunia* (Castel, 2009). *EsSVP* overexpression in *Petunia* shares some phenotypes with that of overexpression of *Petunia UNS*, a *SOC* homolog in *Arabidopsis. 35S*::*UNS* in petunia leads to not fully opened and greener corollas until full maturity and a stem-like structure at the base of the septum inside the elongated ovaries (Ferrario et al., 2004). The phenotype of *35S::EsSVP* transgenic petunia plants also includes shortened style, a stem-like structure at the base of the septum in a significantly longer ovaries, a sunken surface on the top of the stigma, persistent petals on the completely matured fruit (**Figure 6C**). When introduced in *exp*, *35S::EsSVP* could not rescue the mutant phenotype. Like in *Arabidopsis*, differences in the proteins or the 35S promoter expression pattern might be accountable for the lack of complementation.

Recent study showed that SVP delays flowering by repressing integrator gene expression as well as the plant growth regulator gibberellin (GA) biosynthesis. The results link GA biosynthesis to the established regulatory cascade of flower development and illuminate one of the mechanisms by which levels of growth regulators are synchronized with floral transition (Andrés et al., 2014). Regardless of the underlying mechanism, *EsSVP* effected different phenotypes in different genotypes (*Arabidopsis* and petunia). Abnormal flower development was caused by ectopic expression of *EsSVP*, affecting flower and primordia development during the reproductive phase.

## Author Contributions

ZL designed the research, performed vector construction, plant transformation, histological section and wrote the manuscript. YL and SZ performed detection of mutations, gene isolation and expression. ZL and ES contributed reagents and materials. ES performed critical editing of the manuscript. All authors reviewed the final manuscript.

## Conflict of Interest Statement

The authors declare that the research was conducted in the absence of any commercial or financial relationships that could be construed as a potential conflict of interest.

## Footnotes

1 http://www.ncbi.nlm.nih.gov.
