# Supplementary material for: Leaf-Like Sepals Induced by Ectopic Expression of a SHORT VEGETATIVE PHASE (SVP)-Like MADS-Box Gene from the Basal Eudicot Epimedium sagittatum
Source: Front Plant Sci. 2016 Sep 28;7:1461. doi: 10.3389/fpls.2016.01461 (PMC5039176; doi:10.3389/fpls.2016.01461)
Supplement: Supplementary file 1 [file Data_Sheet_1.DOC]

**Supplementary material**

**Supplementary Table S1. List of primer pairs used in this study.**

| **Primer Name Primer Sequence** | | **Description** | |
| --- | --- | --- | --- |
| GSP3-5 | GCAACCTCAGCATCACAAAGAATAGAAAGC | | 5’-RACE |
| GSP5-3 | GCTTTCTATTCTTTGTGATGCTGAGGTTGC | | 3’-RACE |
| *EsSVP*-V-F  *EsSVP*-V-R | AAGATAAGAAGAATGGCAAGGGAG (#6229)  TAAGTCAAGATGTTCACAGGGACG (#6230) | | Full-length isolation  and RT-PCR |
| *EsSVP-*qF  *EsSVP-*qR | GATGGAAGAAAATGAGCGATTG (#6269)  GAAGAGGACCGCCTGAACTG (#6270) | | qRT-PCR |
| *SVP*-qF  *SVP*-qR | CAAGGACTTGACATTGAAGAGCTTCA (#6391)  CTGATCTCACTCATAATCTTGTCAC (6392) | |
| *CO*-qF  *CO*-qR | TGCAAACCCACTTGCT  CCTCCTTGGCATCCTTATCA | |
| *EsActin*-F  *EsActin*-R | TACGAACAGGAGCTGGAGACTT  GATGGTCCAGACTCGTCATACTC | |
| *FT*-qF  *FT*-qR | GGAACAACCTTTGGCAAT  AGCCACTCTCCCTCTGACAA | |
| *FLC*-qF  *FLC*-qR | AGCCAAGAAGACCGAACTCA  TTTGTCCAGCAGGTGACATC | |
| *SOC1*-qF  *SOC1*-qR | AATTCGCCAGCTCCAATATG  CCTCGATTGAGCATGTTCCT | |
| *LFY*-qF  *LFY*-qR | ATTGGTTCAAGCACCACCTC  CAAGAAGCTCCCAACGAAAG | |
| *AP1*-qF  *AP1*-qR | GCAAGCAATGAGCCCTAAAG  ACTGCTCCTGTTGAGCCCTA | |
| *TUB2*-qF  *TUB2*-qR | ATCCGTGAAGAGTACCCAGAT  AAGAACCATGCACTCATCAGC | |
| *PhActin*-qF  *PhActin*-qR | TGCACTCCCACATGCTATCCT (#5922)  TCAGCCGAAGTGGTGAAAGAG (#5923) | |
| *PhActin*-F  *PhActin*-R | AGATCTGGCATCATACCTTCTACA (#58a)  CCMGCAGCTTCCATRCCAATCA (#59a) | | RT-PCR |


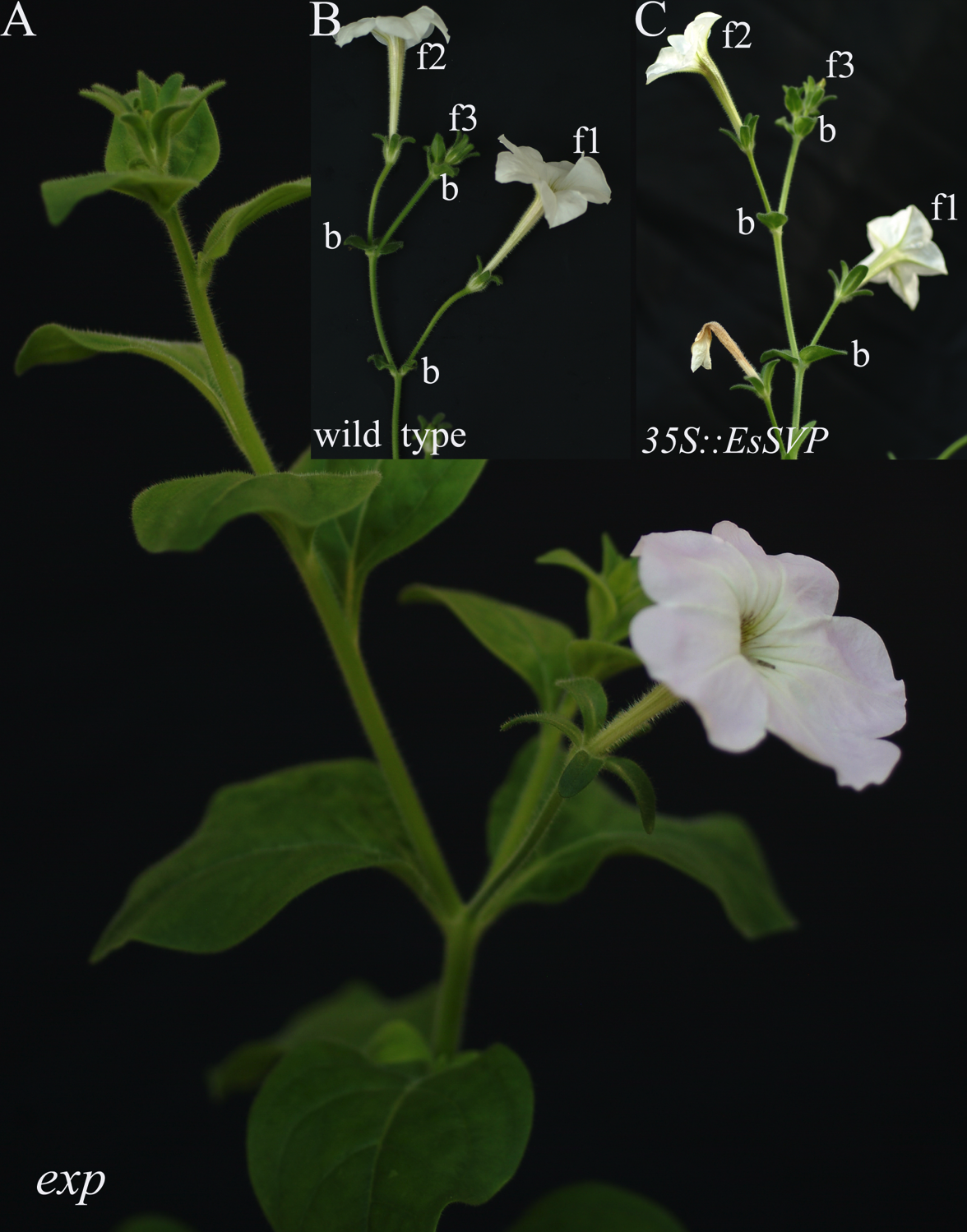


**Supplementary Figure S1. Recessive mutations in *EXP* inhibit cymose branching in the inflorescence.** (A) The single terminal flower in *exp.* (B-C) Three successive terminal flowers in the wild type (B) and *35S::EsSVP* (C). f, flower; b, bract.

1 *GAACACAGTTGATAGAAACTGAGCTAAACCAACTAACTCCTCCCCCAAAACTGGTAAAAAGCAAGGTTGTATGAGAAGATAAAAAGA*

***EsSVP*-V-F**

M A R E K I Q I K K I V N P T A R E V T F S K R R R G L F

88 ATG*GCAAGGGAG*AAGATTCAGATCAAGAAGATTGTCAACCCAACTGCAAGGGAAGTGACCTTCTCTAAGAGGAGAAGAGGGCTTTTC

K K T E E L S I L C D A E V A L I I F S A T G K L F E Y S

175 AAGAAGACTGAAGAGCTTTCTATTCTTTGTGATGCTGAGGTTGCACTCATCATCTTCTCTGCTACCGGGAAACTCTTTGAGTACTCC

**GSP5-3/ GSP3-5**

S S S M K E I L E W H N M H S K N L Q K L D Q P S L E L Q

262 AGCTCCAGCATGAAGGAGATACTTGAATGGCACAATATGCACTCAAAGAACCTTCAGAAACTGGATCAACCATCTCTTGAGTTGCAG

L E S S N Y T R L S K E V S E K S H Q L R Q M R G E E L Q

349 CTGGAGAGTAGCAACTACACCAGATTGAGCAAGGAAGTTTCAGAGAAAAGCCATCAATTAAGGCAAATGAGGGGAGAAGAGCTACAA

G L N L E Q L Q Q L E K S L E T G L G R V L E T K S E R I

436 GGACTAAACCTTGAGCAGCTGCAACAACTGGAGAAATCACTTGAAACAGGATTGGGTCGTGTGCTCGAAACAAAAAGCGAAAGGATT

L K E I S T L H T K G I Q L M E E N E R L R Q Q M M A I T

523 TTGAAGGAGATCAGTACACTCCACACAAAGGGAATACAGTTGATGGAAGAAAATGAGCGATTGAGACAGCAAATGATGGCGATAACT

***EsSVP-*qF**

N G Q K K P I A L E V H E E A Q G Q S S D S V T N V S S S

610 AATGGTCAGAAGAAACCAATTGCTCTAGAAGTTCATGAGGAAGCTCAAGGCCAGTCATCAGATTCTGTCACCAATGTCTCCAGTTCA

G G P L Q D D D S S D T S L K L G L P C S S *

697 GGCGGTCCTCTTCAAGACGATGACAGCTCAGATACTTCGCTCAAGCTGGGGTTACCTTGCTCAAGTTGA*CTGGAGAGAAACTCTTCA*

***EsSVP-*qR**

*784 TTTAGTTGGAACTATTATAATAAGGGATTTAAAGACGTATGCATGTATGATAAGCCTGGTTAATGGGGACACTTAAGATAACCATCC*

*871 CAGACGCGTATCTTAAGTGTGTGTGAGACATTCGAGACATTCGTCCCTGTGAACATCTTGACTTATGGATTGAATTACGAATAGTGG*

***EsSVP*-V-R**

*958 ATTTCAACTCATCCGTTAATATTATCTGTGAAGTTTAGATTTCTTGTCCAAAAAAAAAAAAAAAAAAAAA*

**Supplementary Figure S2. Full-length cDNA sequence of *EsSVP* and corresponding amino acid sequence**. The nucleotide and deduced amino acid sequences of *EsSVP* (1,027 bp) are shown. The 5’/3’-UTR and poly-A are indicated by italic characters. The start and stop codon are indicated by underline characters in red. The primers used for amplifying the full-length *EsSVP* for vector, the 5’/3’-RACE and the fragment for qPCR are underlined in green, brown and blue.
